# Impact of agricultural farms on the environment of the Puck Commune: Integrated agriculture calculator—CalcGosPuck

**DOI:** 10.7717/peerj.6478

**Published:** 2019-02-19

**Authors:** Lidia Dzierzbicka-Glowacka, Stefan Pietrzak, Dawid Dybowski, Michał Białoskórski, Tadeusz Marcinkowski, Ludmiła Rossa, Marek Urbaniak, Zuzanna Majewska, Dominika Juszkowska, Piotr Nawalany, Grażyna Pazikowska-Sapota, Bożena Kamińska, Bartłomiej Selke, Paweł Korthals, Tadeusz Puszkarczuk

**Affiliations:** 1 Physical Oceanography Department, Ecohydrodynamics Laboratory, Institute of Oceanology of the Polish Academy of Sciences, Sopot, Poland; 2 Department of Water Quality, Institute of Technology and Life Sciences in Falenty, Raszyn, Poland; 3 Academic Computer Centre in Gdańsk, Gdańsk, Poland; 4 Department of Environment Protection, Maritime Institute in Gdańsk, Gdańsk, Poland; 5 Municipality of Puck, Puck, Poland

**Keywords:** Agricultural farms, Nutrient balance, Efficiency, Agriculture calculator, Puck commune, Puck buy, Baltic sea

## Abstract

**Background:**

Leaching of nutrients from agricultural areas is the main cause of water pollution and eutrophication of the Baltic Sea. A variety of remedial actions to reduce nitrogen and phosphorus losses from agricultural holdings and cultivated fields have been taken in the past. However, knowledge about the risk of nutrient leaching has not yet reached many farmers operating in the water catchment area of the Baltic Sea.

**Methods:**

The nutrient balance method known as “At the farm gate” involves calculating separate balances for nitrogen (N), phosphorus (P) and potassium (K). After estimating all the components of the nutrient balance, the total balance for NPK is calculated and the data obtained is expressed as the ratio of total change (surplus) to the area of arable land on a farm. In addition, the nutrient usage efficiency on a farm is also calculated. An opinion poll was conducted in 2017 on 3.6% (*n* = 31) of the farms located in commune of Puck. The total area of the farms including arable and grass land ranged from 5 to 130 ha with an average of 45.82 ha. The arable land was on average 30.79 ha ranging from 4.45 to 130 ha while the grassland averaged 12.77 ha and ranged from 0 to 53 ha.

**Results:**

The average consumption of mineral fertilizer in the sample population of farms was 114.9 kg N, 9.3 kg P, and 22.9 kg K·ha^−1^of agricultural land (AL), respectively. N balance in the sample farms being ranged from −23.3 to 254.5 kg N·ha^−1^AL while nutrient use efficiency ranged from 0.40% to 231.3%. In comparison, P surplus in the sample farms was 5.0 kg P·ha^−1^AL with the P use efficiency of 0.4–266.5%.

**Discussion:**

Mean N fertilizer consumption in the tested farms was higher than the average usage across Poland and in the Pomeranian Voivodeship. However, mean consumption of potassium fertilizers was lower than mentioned averages. Mean P fertilizer consumption was higher than in the Pomeranian Voivodeship, but lower compared to the entire country. Generally, on the basis of designated research indicators of farm pressures on water quality, concentrations of total nitrogen and total phosphorus were obtained. CalcGosPuck (an integrated agriculture calculator) will help to raise farmers’ awareness about NPK flow on farm scale and to improve nutrient management.

## Introduction

Leaching of nutrients from agricultural areas is the main cause of water pollution and eutrophication of the Baltic Sea. A variety of remedial measures to reduce nitrogen (N) and phosphorus (P) losses from agricultural holdings have been taken in the past. However, knowledge about the risk of nutrient leaching has not yet reached many farmers operating in the watershed areas of the Baltic Sea. Nevertheless, the growing international consciousness on the need for water quality improvement has influenced the desire to expand knowledge and social awareness of environmental implications of water quality worldwide. There are relatively cheap and simple prevention measures (e.g., crop rotation, soil fertility analysis, separation of pastures from water courses and reservoirs or systematic on-farm Advisory Services), but not all of them have been implemented or entered into the list of 25 priority measures set out within the framework of the Baltic Compass project (Salomon & Sundberg, 2012). One of the reasons for this is that these measures should be worked out in practice by farmers based on their knowledge, and then adapted to the given farming conditions (Ulén, Pietrzak & Tonderski, 2013).

The farm is the basic organizational unit in agriculture and it produces food and raw materials for industry. Production involves a large quantity of nutrients, only a fraction of which is converted into animal and vegetable products. The surplus of the unused nutrients in production accumulates in the soil, or are lost to surface waters, drain water, groundwater, or to the atmosphere. The loss of nutrients is an economic cost in terms of reduced production obtained with higher cost of inputs and poses a threat to the environment. N and P compounds are of special concern in environmental quality management because they are lost through several pathways such as surface runoff, subsurface flow and leaching within soils, water and wind erosion, emissions of gaseous forms of N and their deposition by atmospheric precipitation (Pietrzak, 2013).

Arguably, nutrient losses are inevitable; however, given their environmental and the economic impacts on production and environmental quality, they should be kept below acceptable minimum thresholds. Therefore, it is essential to create farm production thresholds to ensure effective nutrient management. The “At the farm gate” method is one way to conduct a nutrient balance for a farm. This method is a good educational and decision support tool in the area of agricultural production activities, for such entities as farmers, agricultural advisors, agricultural school and university teachers as well as employees of state and local government institutions who are responsible for agri-environmental policy implementation. It is particularly important for farmers and agricultural consultants and advisors cooperating with them. In this partner system, the “At the farm gate” method is used as a measure that could potentially improve the efficiency of fertilizer components management in an agricultural holding, which is a beneficial factor for both economic and environmental reasons. Therefore, farmers and agricultural advisors must be trained to acquire the knowledge and skills to estimate nutrient balances (Pietrzak, 2013). However, there are best practices for increasing nutrient use efficiency in order to reduce expenditure on fertilizers use and feed in commercial agriculture. These entail use of computer programs to estimate nutrient balances in the farm especially for NP. In the United Kingdom, for example, the software for calculating the “At the farm gate” nutrient balance is available free of charge for farmers and agricultural advisors as a module of the planning land applications of nutrients for efficiency and the environment (PLANET) system (Farmgate Nutrient Balance Help file). In Sweden, a computerized NPK balancing system called “Greppa Näringen, that is, Focus on nutrients” was implemented on a large scale (C. Nilsson, 2016, personal communication) and used by farmers in cooperation with agricultural advisors on a voluntary basis and makes significant impact (Jakobsson, 2012). Furthermore, in the United States, the application for balancing fertilizer components on the farm was disseminated nationwide as part of the “livestock and poultry environmental stewardship” program (Koelsch, 2002; Koelsch & Franzen, 2002).

The research presented in this paper was conducted as part of the project on modeling of the impact of the agricultural holdings and land-use structure on quality of water in the Bay of Puck—Integrated information and forecasting Service “WaterPUCK” (Dzierzbicka-Głowacka et al., in press).

**Figure 1:**
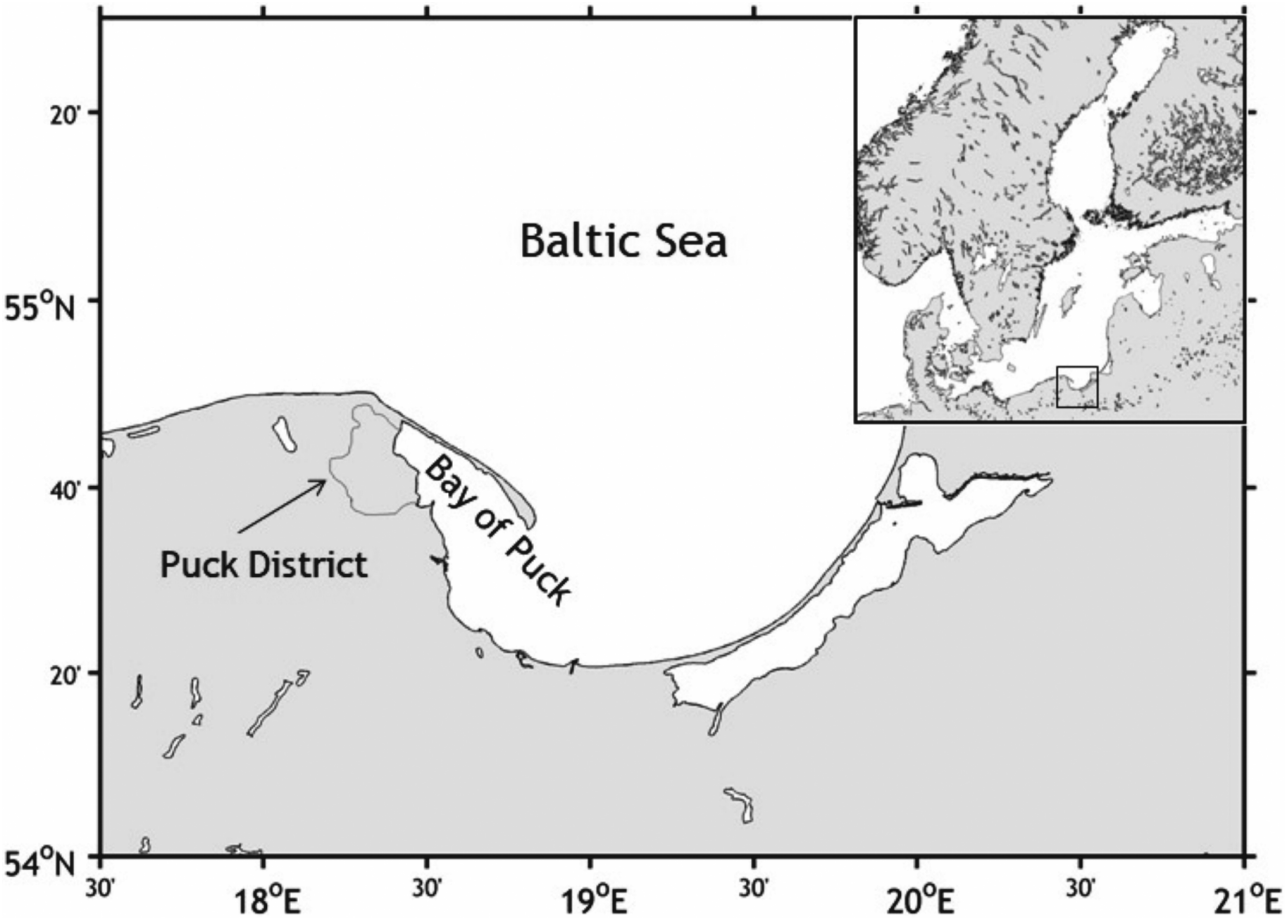
Map of the study area: Puck District and Bay of Puck. The Bay of Puck, southern Baltic Sea is an example of a region that is highly vulnerable to anthropogenic impact. Therefore, it has been included into Natura 2000.

The purpose of the project was to determine the current and future environmental status of surface water and groundwater quality in the Puck Commune and its impact on the Bay of Puck environment (Fig. 1). The most significant input of nutrients and pesticides in the environment comes from agricultural source and surface structure usage, for an example, sewers or drainage ditches. Therefore, objective of the project was to estimate the impact of nutrient loading by compiling the recent knowledge, factoring in the essential in situ measurements, and using advanced modeling.

## Material and Methods

### Integrated agriculture calculator—CalcGosPuck

The web tools obtained within the project (service WaterPUCK with CalcGosPuck) were modified account for many innovative measures, processes and models to provide a basis for the “green economy” development that could be implemented in other Baltic Sea catchment areas. This is in line with the objectives of European legislation, including: (i) the Nitrates Directive (Council Directive 91/676/EEC, 1991), (ii) the Water Framework Directive (2000/60/EC), (iii) the Marine Strategy Framework Directive (2008/56/EC) and (iv) the Habitats Directive (92/43/EEC) as well as with the HELCOM Baltic Sea Action Plan and the strategic program of environmental protection for the Puck Commune.

**Figure 2:**
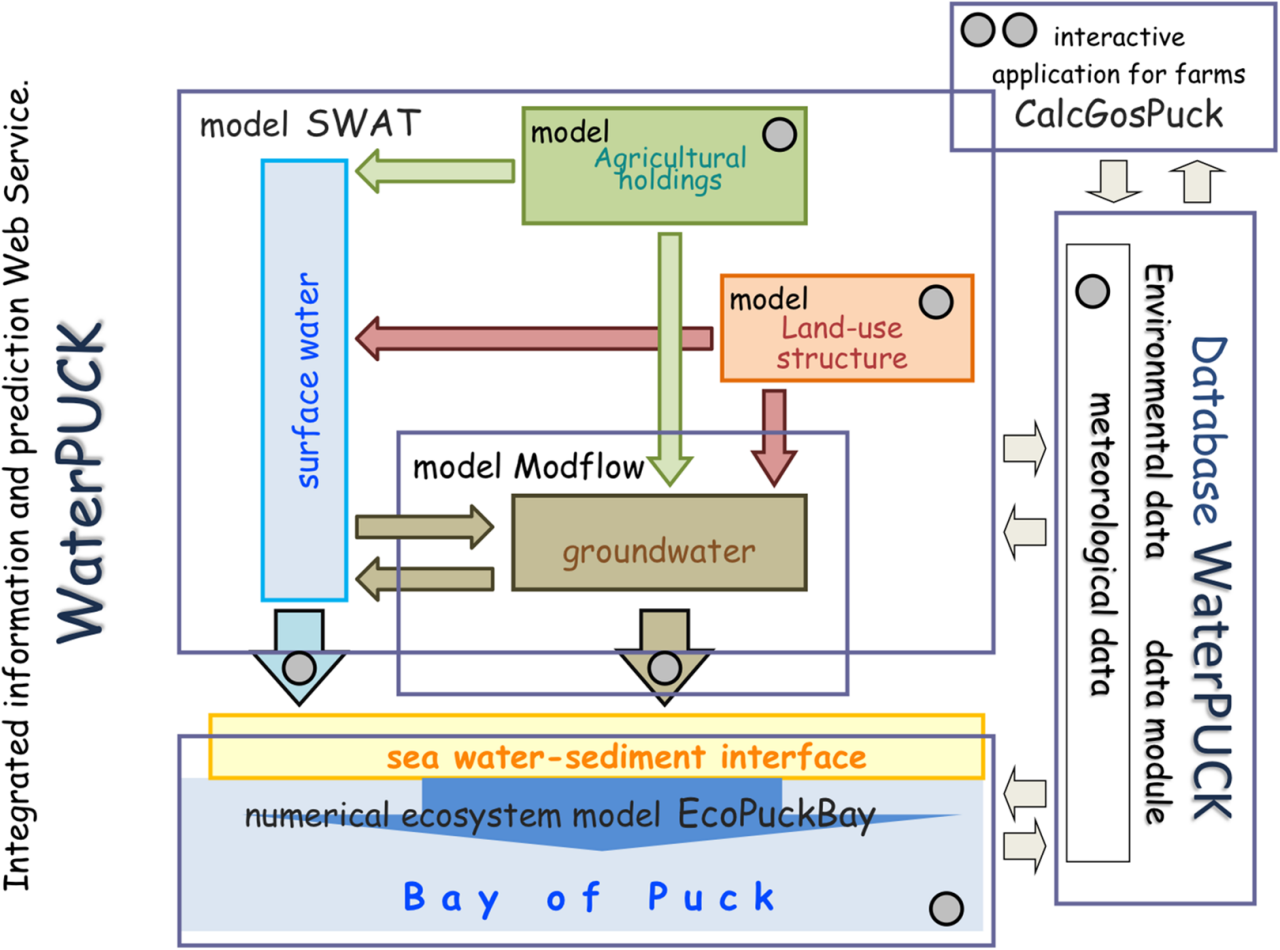
The shame of the WaterPUCK service. Integrated information and prediction Service WaterPUCK includes surface water model (based on SWAT Soil and Water Assessment Tool), groundwater flow model (based on Modflow code), 3D environmental model of the Bay of Puck EcoPuckBay (based on the POP code and 3D CEMBS model of the Battic Sea) and an integrated agriculture calculator called “CalcGosPuck” plus large Database WaterPUCK.

The WaterPUCK service (Fig. 2) includes the following: a surface water model based on SWAT, a groundwater flow model “GroundPuck” based on Modflow, a 3D environmental model of the Bay of Puck “EcoPuckBay” based on the POP code and an integrated agriculture calculator called “CalcGosPuck.” The CalcGosPuck, presented in this paper, was developed as the first module of the WaterPuck service. Data obtained from farms and defined in this model were used to determine fertilizer components loads released from agricultural production to the environment, including surface and groundwater.

### The general concept of nutrient balance on farms

**Figure 3:**
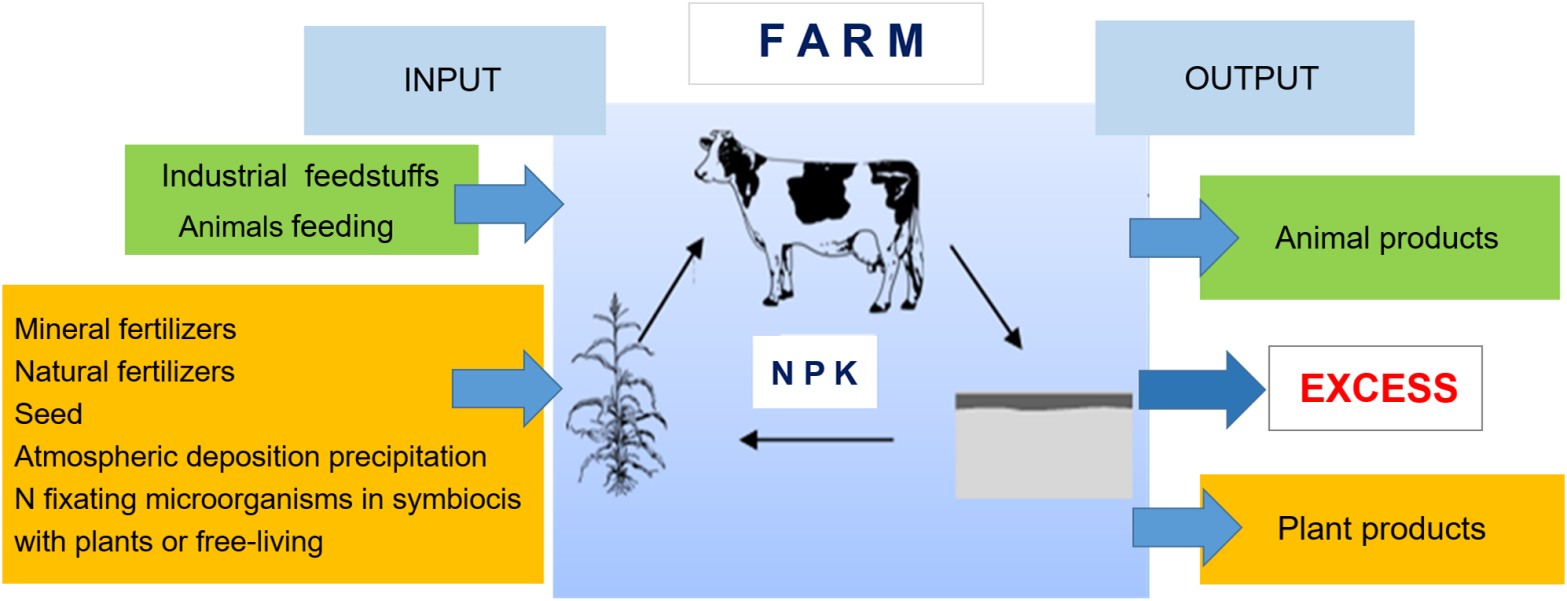
Schema of the nutrient balance method “At the farm gate”; own elaboration (Pietrzak, 2013).

The “At the farm gate” nutrient balance method usually involves calculating separate balances for NPK nutrient elements. The principle is the same for all three nutrients, with the exception that the N balance sheets include more factors because of larger number of N nutrient sources into the farms (e.g., legumes crops, deposition from the atmosphere). The procedure for establishing balance of nutrients using the “At the farm gate” method has been described in detail by Pietrzak (2013). Preparation of the nutrient balance using “At the farm gate” method involves determination of input and output streams on the farm (Fig. 3).

The mass of nutrients imported onto a farm is calculated as the amount of input in: (i) mineral fertilizers (own study based on data producers of mineral fertilizers); (ii) purchased concentrated fodders (Mercik, 2002); (iii) purchased bred animals (Fagerberg, Salomon & Steineck, 1993; Wrzaszcz, 2009; Rutkowska, 2010; Szewczuk, 2010); (iv) natural fertilizers (farm-produced or externally purchased manure) (Maćkowiak, 1997; Grabowski, 2009); (v) other purchased products (Fagerberg, Salomon & Steineck, 1993; Wrzaszcz, 2009; Rutkowska, 2010; Szewczuk, 2010); (vi) atmospheric deposition (adopted for the Pomeranian Voivodeship) (data from Institute of Meteorology and Water Management - National Research Institute); vii) symbiotically fixed nitrogen (Schmidtke, 2008; Høgh-Jensen et al., 2004); (viii) nitrogen introduced by free-living soil microorganisms (Mazur, 1991); while the masses of nutrients exported from the farm are calculated as the amount of output in sold animal and plant products (Fagerberg, Salomon & Steineck, 1993; Wrzaszcz, 2009; Rutkowska, 2010; Szewczuk, 2010).

### Estimating nutrient balance and usage efficiency

After estimating all the components of the nutrient balance, the total balance (surplus or deficit) for N, P and K was calculated as a difference between inputs and outputs. The data obtained was then expressed as a ratio of total change to area of agricultural land (AL) on the farm and the nutrient usage efficiency on the farm was calculated. The use efficiency of NPK is the ratio of the amount leaving the farm (outputs in plant and animal products, not including leaching, volatilization) to the amount entering the farm (inputs) expressed as a percentage. The nutrient usage efficiency was then used to define the percentage of nutrients brought into the farm, which are used directly for production.

Analysis of the correlation between N and P surplus and selected elements of the balance of these components was carried out using the STATISTICA 7 Soft. The nonparametric method of calculating the Spearman rank correlation coefficient was used, because the data was not normally distributed (Spearman, 1904).

## Farms in the Puck Commune

### Agricultural lands and livestock production

An opinion poll was conducted on 31 farms within the Commune of Puck, which is approximately 3.6% of all farms in this Commune. The average area of the farms is 45.82 ha with a range of 5–130 ha including arable land. The average area of arable land is 30.79 ha with a range of 4.45–130 ha while the mean area of grassland is 12.77 ha ranging from 0 to 53 ha (Fig. S1).

**Figure 4:**
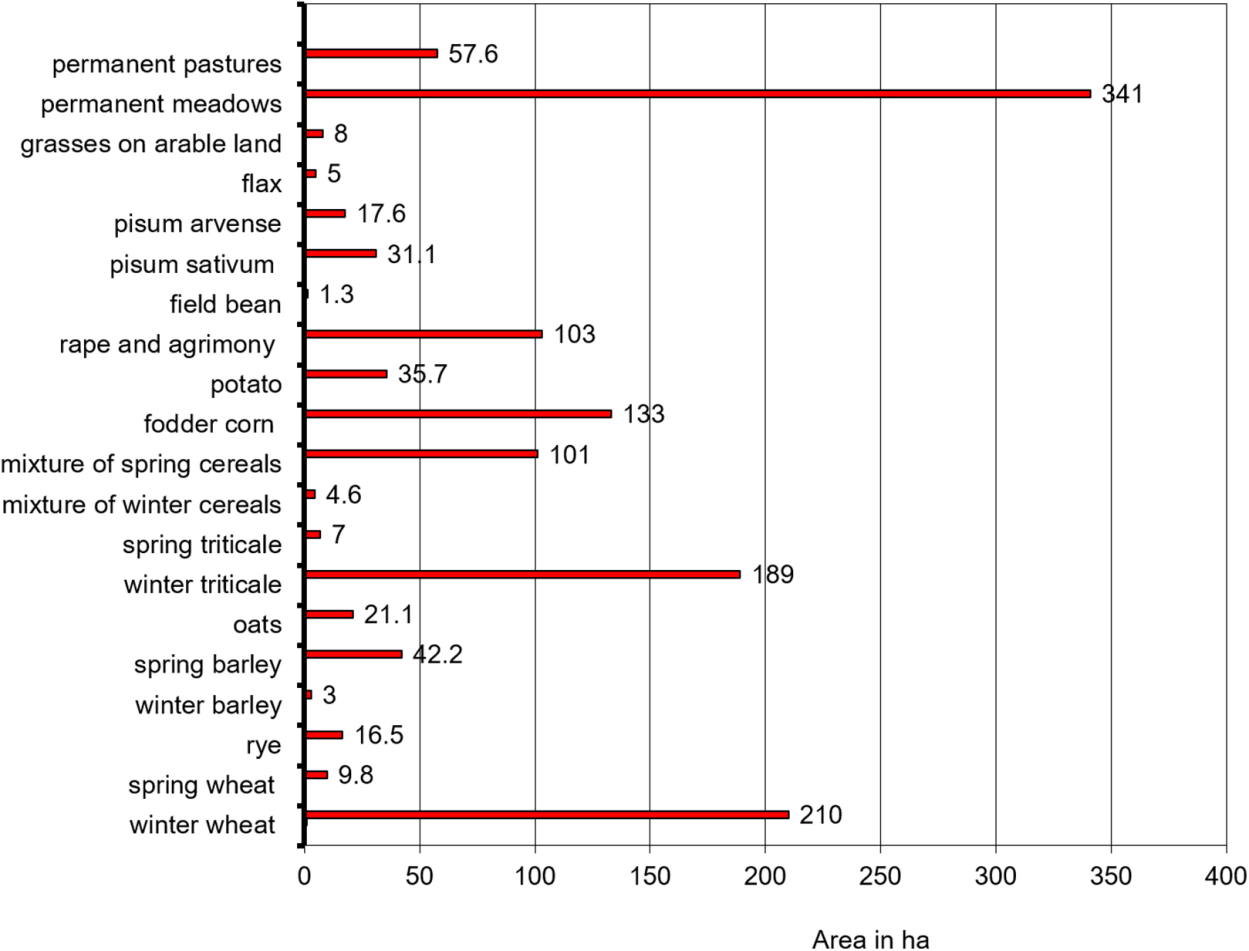
Type and area of arable land or grassland in farms participating in the WaterPUCK project.

Within the test area of the AL, the majority of soils (90.3%, *n* = 28) are medium -Category III (21–35% content of particles with diameter less than 0.02 mm) (Jadczyszyn, Niedźwiecki & Debaene, 2016). The soils in the remaining farms (9.7%, *n* = 3) include light texture soils (11–20% content) (Fig. S2). The types and areas of the field-scale crops and grasslands in farms participating in the WaterPUCK project are given in Fig. 4, and animal population, type, and the barn maintenance systems are given in Table 1.

**Table 1:** Animal population, type and the maintenance system in study farms of Puck Commune.

Farm Code	Farm area (in ha)	Profile of the animal production	Stocking density		Production of nitrogen in natural fertilizers		
			LU	LU ha^−1^	Animals maintenance system	kg N	kg N ha^−1^
1	48	Milk and beef livestock	51.3	1.1	Shallow litter	2,308	48
3	81	Milk and beef livestock	85.6	1.1	Shallow litter	3,843	48
4	17.3	Beef and pork livestock	18.4	1.1	Shallow litter	495	27
5	51.5	Beef and pork livestock	15.4	0.3	Shallow litter	917	18
6	16	Milk and beef livestock	14.3	0.9	Shallow litter	772	48
7	38.2	Beef livestock	21.2	0.6	Shallow litter	723	19
9	70	Milk and beef livestock	70.3	1.0	Shallow litter	3,192	46
10	29.5	Milk and beef livestock	47.3	1.6	Shallow litter	1,899	64
11	18	Beef and pork livestock	8.3	0.5	Shallow litter	422	24
13	43	Pork livestock	52.4	1.2	Shallow litter	3,402	79
14	10.5	Pork livestock	2.9	0.3	Shallow litter	214	28
15	100	Milk and beef livestock	61.6	0.7	Shallow litter	2,662	30
18	77.5	Pork livestock	67.6	0.8	Litter free	4,449	56
19	120	Milk and beef livestock	148.6	1.2	Shallow litter	6,527	54
20	45	Beef livestock	34.4	0.8	Shallow litter	1,171	26
21	15	Pork livestock	45.0	3.0	Shallow litter	2,073	138
22	62	Milk and beef livestock	36.6	0.6	Shallow litter	1603	26
23	36	Milk and beef livestock	24.0	0.7	Shallow litter	1,095	30
24	7.24	Pork livestock	5.42	0.8	Shallow litter	349	48
26	118	Milk and beef livestock	45.5	0.4	shallow litter	4,716	40
27	19	Farming and horse breeding	24.7	1.3	Shallow litter	836	40
28	38	Milk and beef livestock	41.9	1.1	Shallow litter	1,828	48
29	16.5	Milk and beef livestock	34.9	2.1	Deep/shallow litter	2,385	145
30	5.0	Pork livestock	6.4	1.3	Shallow litter	398	80
31	13	Beef and pork livestock	1.3	0.01	Deep litter	70	5

The profile of production systems in the study farms is presented in Table 2.

**Table 2:** The profile of production systems in the study farms in the Puck Commune.

Production system	No. of farms	Proportion of total (%)
Milk and beef	12	38.7
Pork only	6	19.4
Pork and beef	4	12.9
Beef only	2	6.5
Horse breeding	1	3.2
None^1^	6	19.4

**Note:**

1 No livestock production (plant production only).

In the majority of farms (96.8%, *n* = 30) the management system of livestock manure was the slurry and solid manure system, in which animals are maintained in livestock buildings on a shallow litter. An exception was the farm marked Code 29, where some of the young animals (calves and heifers) were kept in deep leaf litter, and one small farm (Code 31) where all the animals (calves and piglets) were kept in a deep barn, in a total of 1.3 of livestock unit (LU). The livestock density was variable ranging from

- 0.1–1.0 LU·ha^−1^ on 14 farms;
- 1.1–2.0 LU·ha^−1^ on nine farms; and
- 2.1–3.0 LU·ha^−1^ on two farms.

In the high density farms (c) the mass of nitrogen produced in natural fertilizers per hectare was relatively high, with values ranging from 138 to 145 kg N·ha^−1^. However, it did not exceed the limit of land application of 170 kg N·ha^−1^ per year stated in the Nitrates Directive.

In a small portion of the farms (Codes: 9, 11, 20 and 23) involved in the production of milk and beef livestock, animals have periodically been at pasture. The farm marked Code 27, which breeds and raises horses, has also been using pastures.

### Crop rotation and after-crops

Out of the Puck Commune farms surveyed, the vast majority of them (96.8%, *n* = 30) practice crop rotation. The most common (76.6%, *n* = 23) kind of crop rotation was cereal rotation (the share of cereal plants above 60%). The most distinctive types of cereal rotation were silage maize-winter wheat-spring grain mixtures, winter wheat-spring wheat-winter wheat-oat and spring barley-oat-spring grain mixtures-potatoes.

The most relevant rotation was field-corn cereal (above 60%), on 23 farms (76.7%).

Only 19.4% (*n* = 6) out of all farms use after-crops (a later crop of the same year from the same soil). In farms with additional vegetative cover two types of after-crops—catch crops and mixed cropping (companion crops)—have been equally preferred. These after-crops were in the majority of cases (83.3%, *n* = 5) incorporated in green manure. The cultivated area with after-crops ranged from 14.4% to 35.7% of farms’ total arable lands.

### Storage of natural fertilizers and silage

In all sample farms all structures used for the storage of manure regardless of size meet the requirements of Polish legislation “Action program aimed at reducing the outflows of nitrates from agricultural sources” (Ministry of Agriculture and Rural Development of Poland, 2007) for minimum distance of 20 m from wells, edges of waterways and reservoirs. Moreover, a large proportion (82.6%, *n* = 19) of the dung panels and tanks for manure are less than 14 years old. Thus, there is a high probability of effectively stopping leachate of manure and slurry leakage (Fig. S3). In three farms manure was stored directly on the ground, but the piles are located on flat terrain where the soil is neither sandy nor waterlogged at a distance of more than 20 m from the edges of waterways and reservoirs. However, one of the farms was obligated to have a slurry storage tank, due to the litter-free system of keeping livestock. On this farm the current tank was made in 2013 and is located at a distance of more than 20 m from the protected zones of water sources and water intakes and the of the edges of reservoirs and waterways. In almost 50% of the farms (*n* = 16), the most common practice to store compacted silage is special plastic bales that limit the risk of silage juice although, about 30% (*n* = 9) silage is stored in field piles directly on the grounds less frequently.

### Permitted dates to use natural fertilizers

In accordance with the Polish law—Act of July 10, 2007 on fertilizers and fertilizing (Ministry of Agriculture and Rural Development of Poland, 2007), natural and organic fertilizers, in either liquid or solid form (manure, liquid slurry, slurry), were allowed to be applied on field between March 1st and November 30th. Permitted dates of solid manure use on arable lands and liquid natural fertilizers use (manure, slurry) on permanent meadows with marked dates of fertilizer uses by farmers in the Puck Commune are given in Figs. S4 and S5, respectively.

## Results

### Integrated agriculture calculator–CalcGosPuck

**Figure 5:**
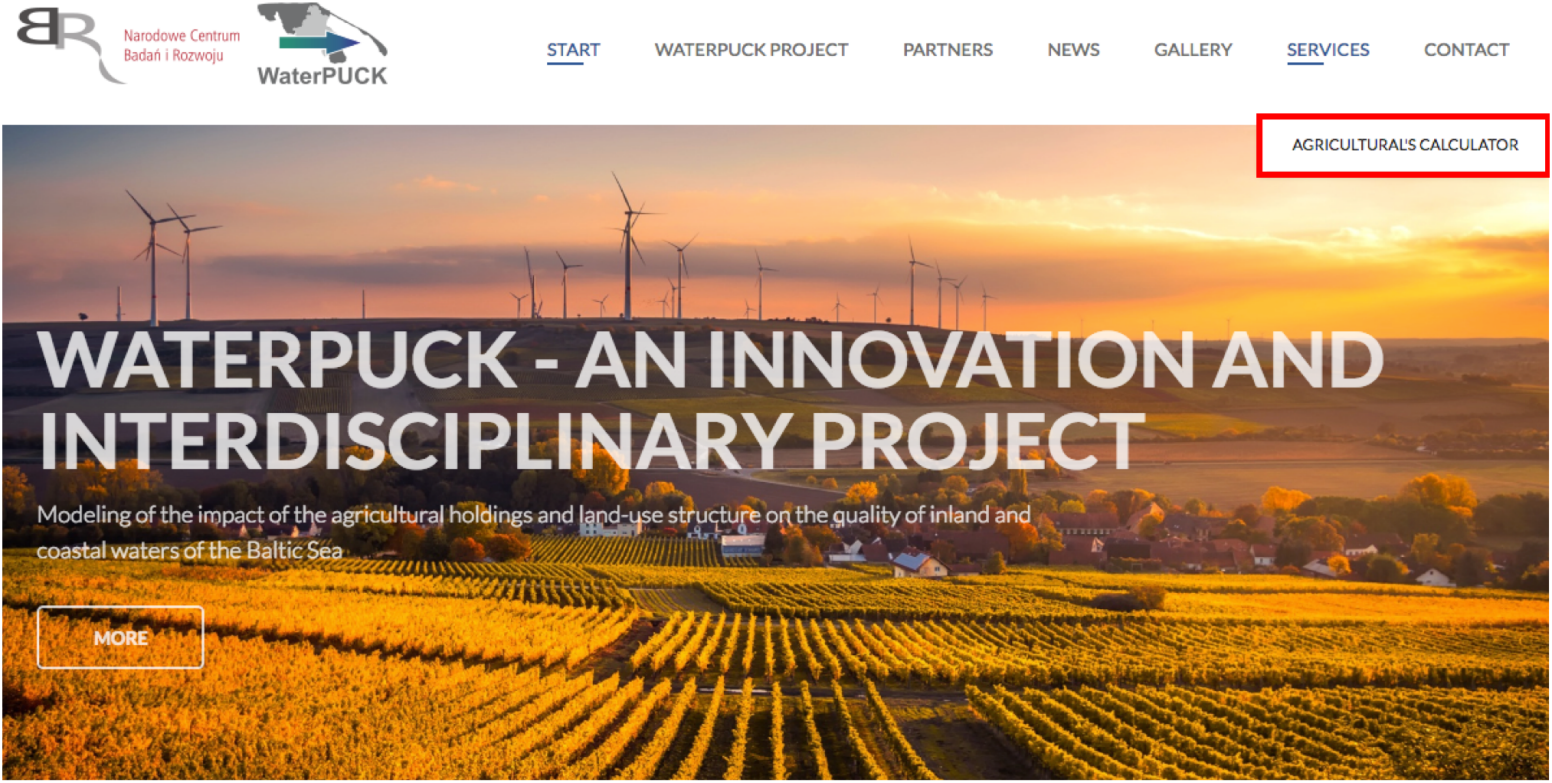
The selection page of the CalcGosPuck agricultural’s calculator.

In accordance with the “At the farm gate” concept method, the agriculture calculator “CalcGosPuck” was developed. The CalcGosPuck calculator works as an independent application designed to calculate the nutrient inputs and outputs, and then the surplus/deficit and the nutrient use efficiency on a farm. The user gives the farm size and selects the required province, input and output products for balance and gives their amount. CalcGosPuck works properly (see the website www.waterpuck.pl in Service—Fig. 5).

**Figure 6:**
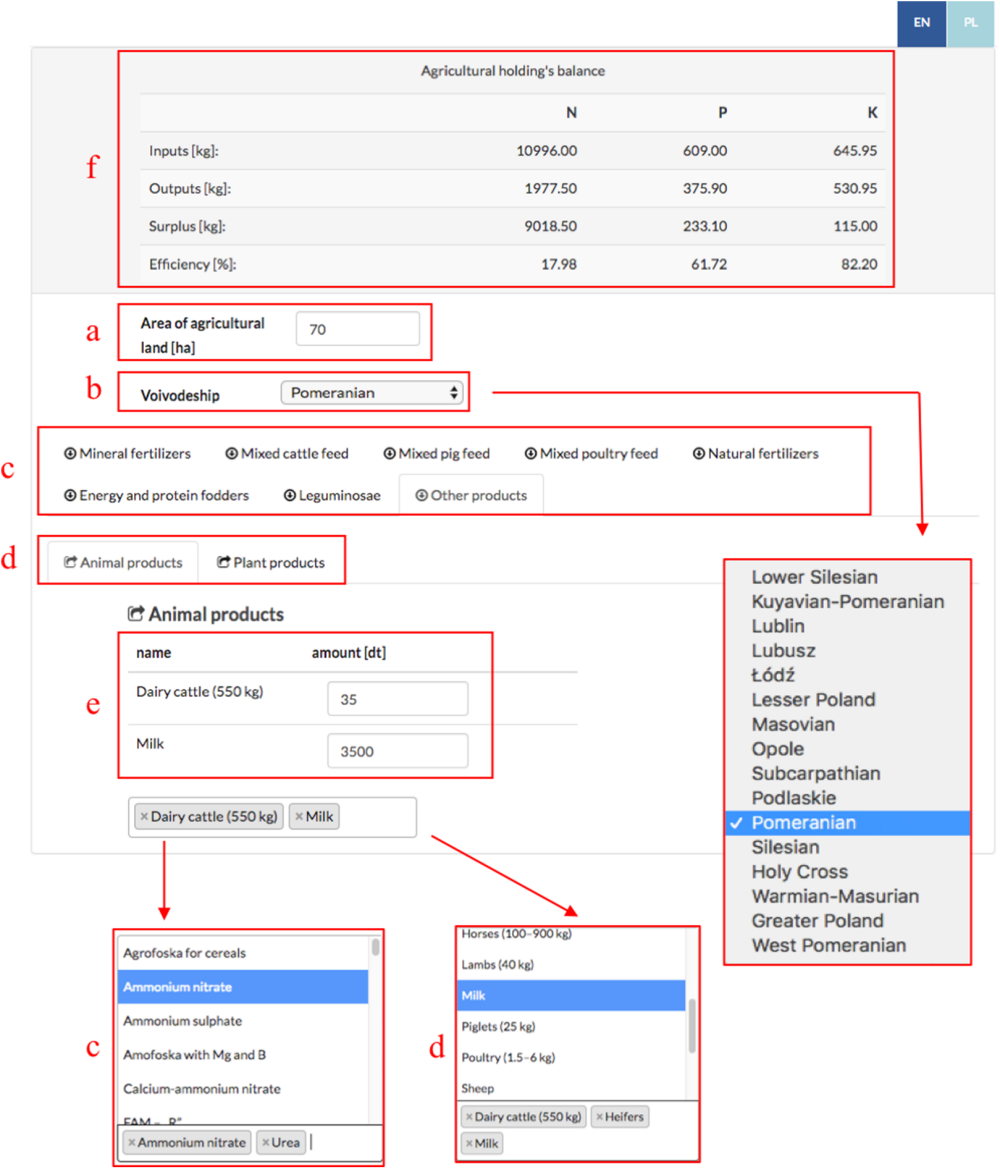
Calculating nutrients balance in farm. Choose parameters for farm: (A) area of agricultural land; (B) Voivodeship; (C) inputs; (D) outputs; (E) amounts of inputs/outputs. The result of the calculations is shown in the table (F).

One should enter specified data (Fig. 6) into the CalcGosPuck calculator in order to determine inputs, outputs, NP surplus (or deficit) and the use efficiency of nutrients on the farm: (i) the area of AL of the farm (in hectares) (Fig. 6A); (ii) the province in which the farm operates (Fig. 6B); (iii) select indicators of what is imported onto the farm (mineral fertilizers, concentrated fodder (mixed cattle feed, mixed pig feed, mixed poultry feed), purchased animal products, natural fertilizers, other purchased plant products, by atmospheric precipitation, by legumes, and fixed by soil microorganisms) (Fig. 6C); (iv) select indicators of what is exported from the farm (in animal and plant products sold) (Fig. 6D); (v) give the amount of each selected indicator (Fig. 6E). After each parameter is selected, the basic data are automatically set down input, output, surplus (or deficit = value with a minus sign) and the data related to the efficiency of the farm are displayed in the top bar (Fig. 6F).

Case Study Application of the Calculator (on the example of a farm marked Code 9)

Step 1: Enter the area of AL [in ha]: 70;
Step 2: Select the Voivodeship: Pomerania;
Step 3: Select inputs and their amounts:
  − in mineral fertilizers: urea = 100 dt, ammonium nitrate = 50 dt,
  − in energy and protein fodders: rape cake for animals = 240 dt, dried pulp = 150 dt, post-extraction soya meal = 400 dt;
  − in other plant and animal products: maize (grain) = 120 dt, heifers = 15 dt;
Step 4: Select outputs and their amount:
  – animal products: milk = 3,500 dt, dairy cattle = 35 dt.
Step 5: Results of the calculations (Fig. 6F):
Budget:
  Inputs: N: 10,996.00 kg; P: 609.00 kg; K: 645.95 kg;
  Outputs: N: 1,977.50 kg; P: 375.90 kg; K: 530.95 kg;
  Surplus: N: 9,018.50 kg; P: 233.10 kg; K: 115.00 kg;
  Efficiency: N: 17.98%; P: 61.72%; K: 82.20%.

### Consumption of natural fertilizers

**Figure 7:**
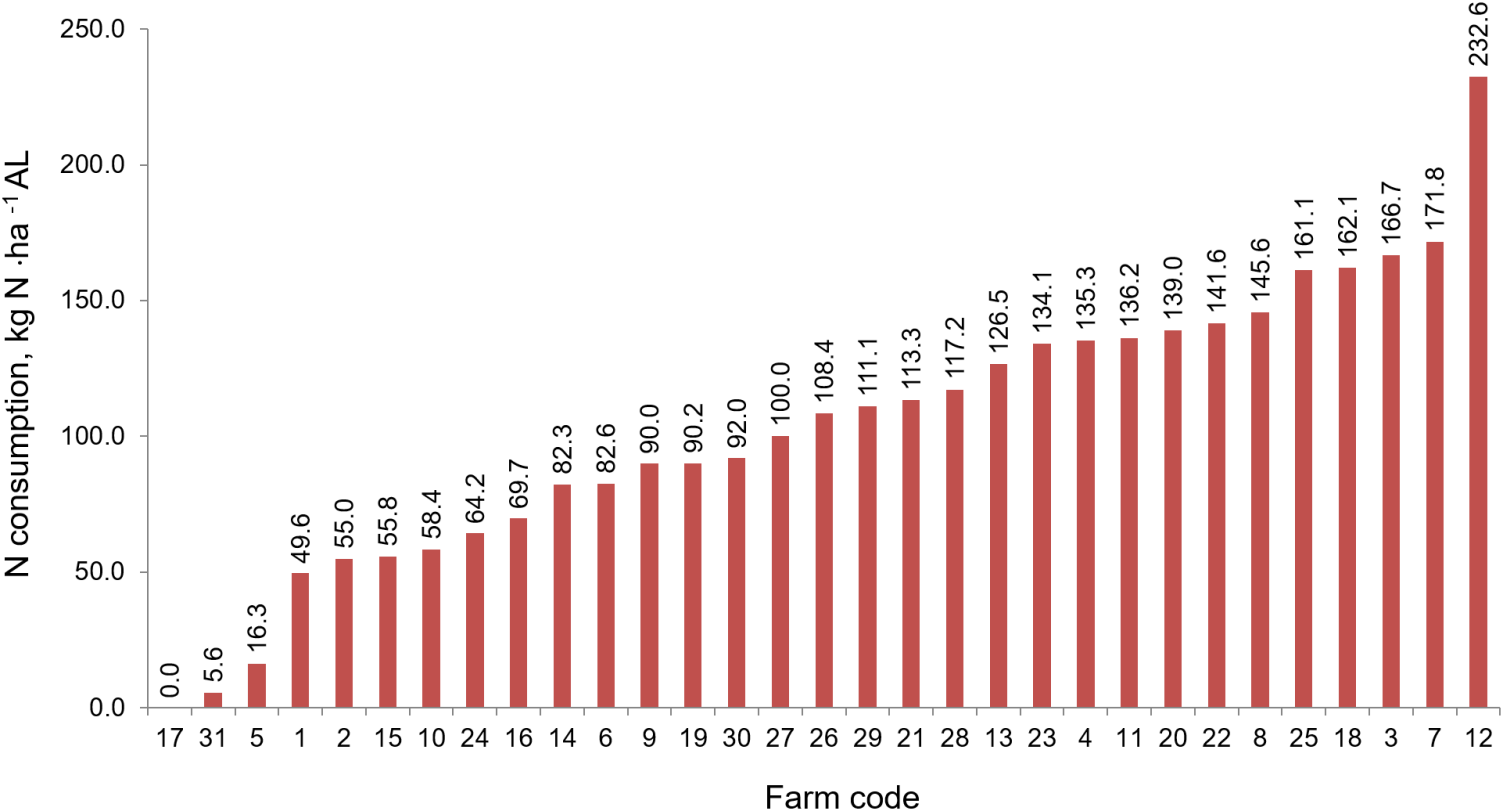
The consumption of nitrogen fertilizers in individual farms in farms participating in the WaterPUCK project.

**Figure 8:**
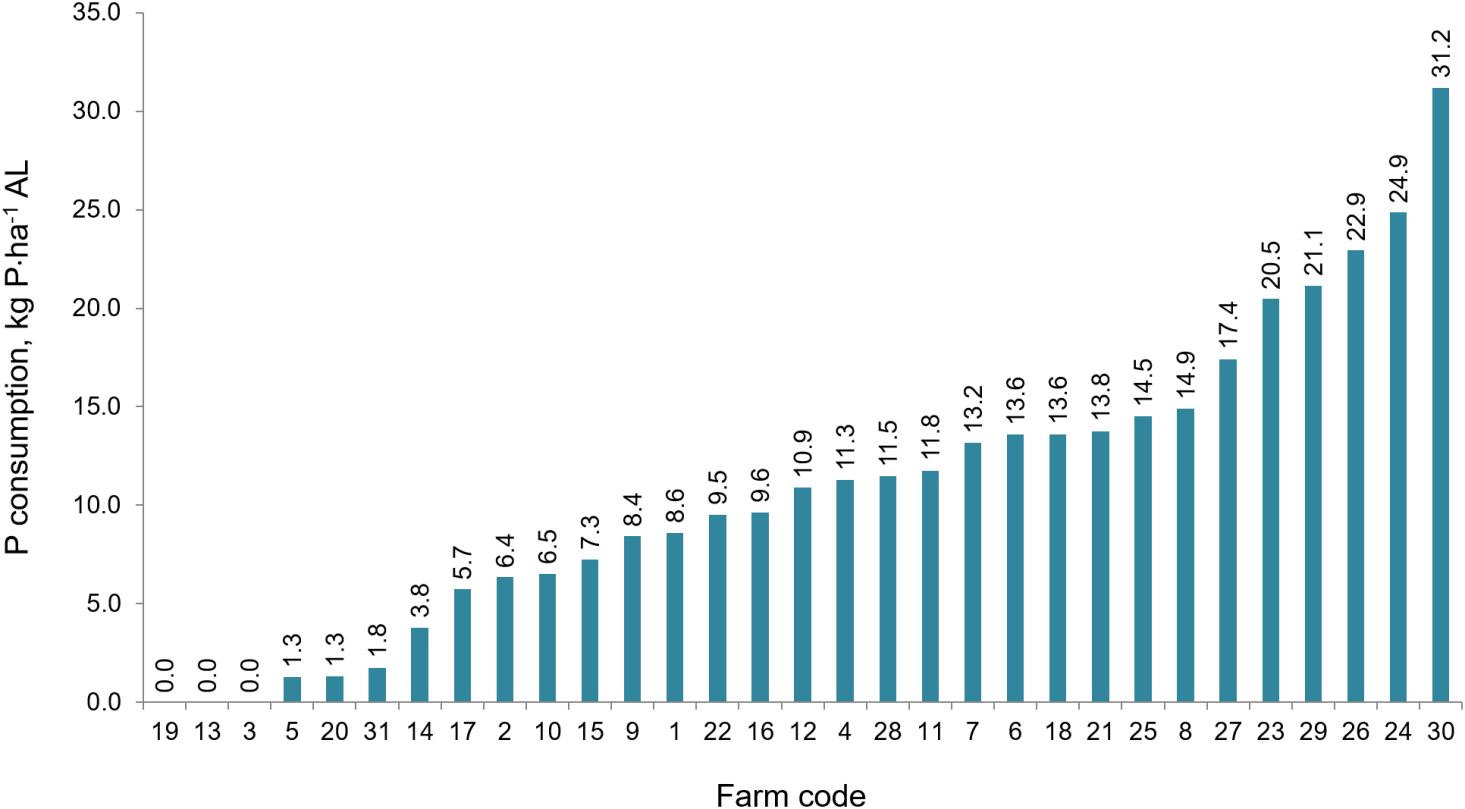
The consumption of phosphorus fertilizers in the individual farms in farms participating in the WaterPUCK project.

**Figure 9:**
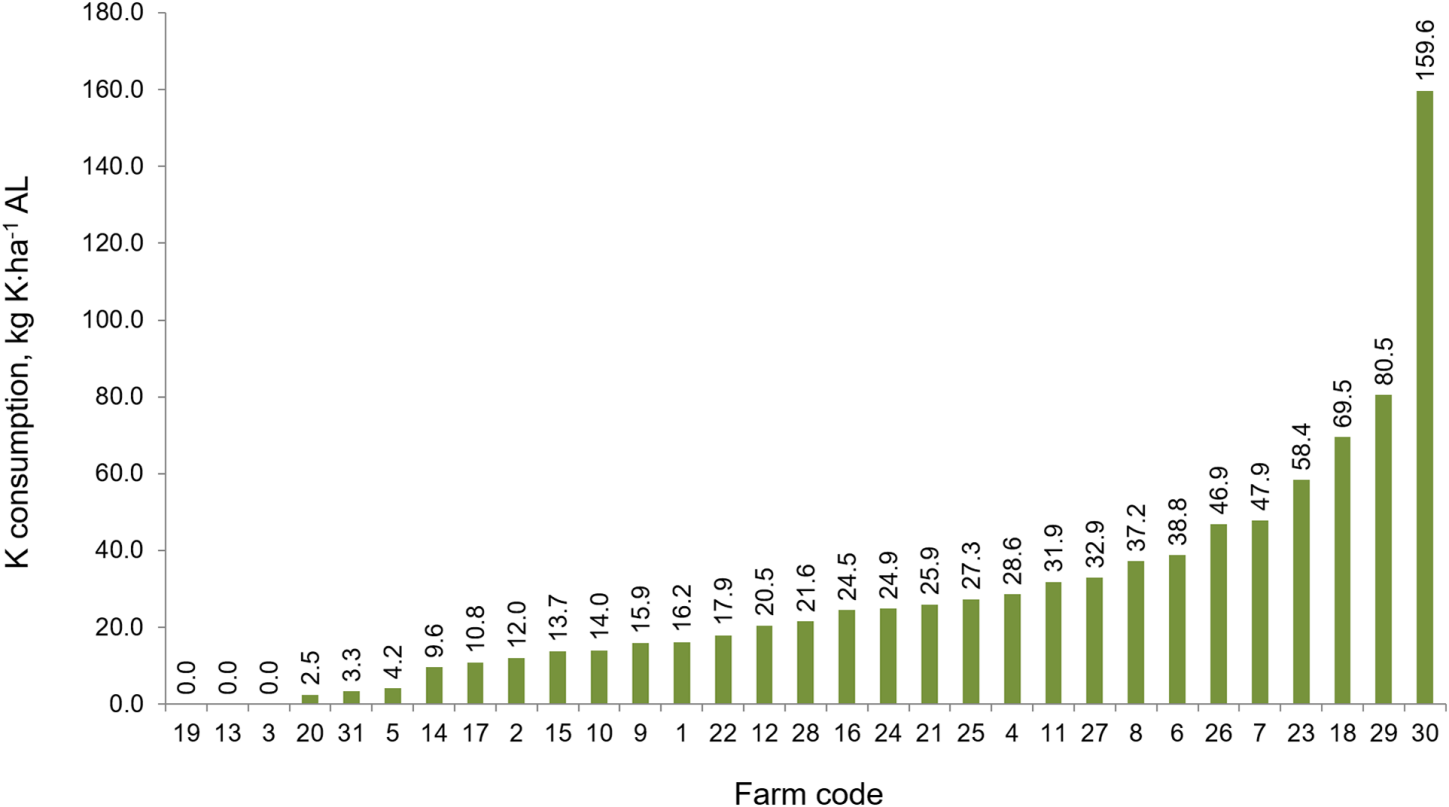
The consumption of potassium fertilizers in the individual farms in farms participating in the WaterPUCK project.

The average consumption of mineral NPK ha^−1^in the study area ranged within the respective levels of: 114.9 kg N, 9.3 kg P, and 22.9 kg K·ha^−1^ AL. On the individual farms, consumption of the components listed was highly variable with a range 0–232.6 kg N·ha^−1^ (Fig. 7); 0–31.2 kg P·ha^−1^ (Fig. 8) and 0–159.6 kg K·ha^−1^ (Fig. 9).

### Environmental aspects of fertilizer usage

With regard to the conditions of fertilizers application, it was determined that:

– On 29 out of the 31 tested farms (93.5%), the annual dosages of nitrogen fertilizers (mineral, natural, organic) were divided into parts during the growing season, usually into three in case of arable lands and two fertilizations of permanent meadows.
– On 19 farms (61.3%) have arable land on parcels with steep slopes (more than 10%). On 16 out of them (84.2%) the general rules of fertilizer usage on steep slopes were taken. In only two agricultural holdings (10.5%) the rules have not been followed. In cases of parcels with a slope of more than 10%, cultivation treatments have been carried out in a direction transverse to the slope leaving the ridge up the slope.
– On two farms (6.5%) fertilizers were applied on field in situations when the soils was flooded, covered by snow or frozen to a depth of 30 cm, and during rainfall. Municipal sewage sludge has not been used in areas of special flood hazard, temporarily flooded and swampy areas, or on high permeability areas on any of the farms.
– On the majority of the tested farms (87.1%, *n* = 27), there were ALs located at a distance of less than 50 m from the edges of waterways and lakes. On the other hand, on most of them (63%, *n* = 17) in the areas close to waterways or reservoirs, fertilization has not been used. In six cases (22.2%) fertilization has been used at a distance less than 20 m from the edges of waterways and lakes.
– Records of agricultural treatments containing information about dates and doses of fertilization were being kept on 23 agricultural holding (74.2%). On the remaining seven farms (22.6%) agro-technical practices were not documented and on one—there were no data.
– Only one of the analyzed farms (3.2%) kept records of natural fertilizers disposal (agreement for sale of any surpluses).
– Nitrogen balance estimation and fertilization plans were being developed on 20 (64.5%) of all the farms. In remaining ones, there were either no balance sheets and fertilization plans or there was no information about that.

### The surplus and use efficiency of nitrogen, phosphorus and potassium

**Figure 10:**
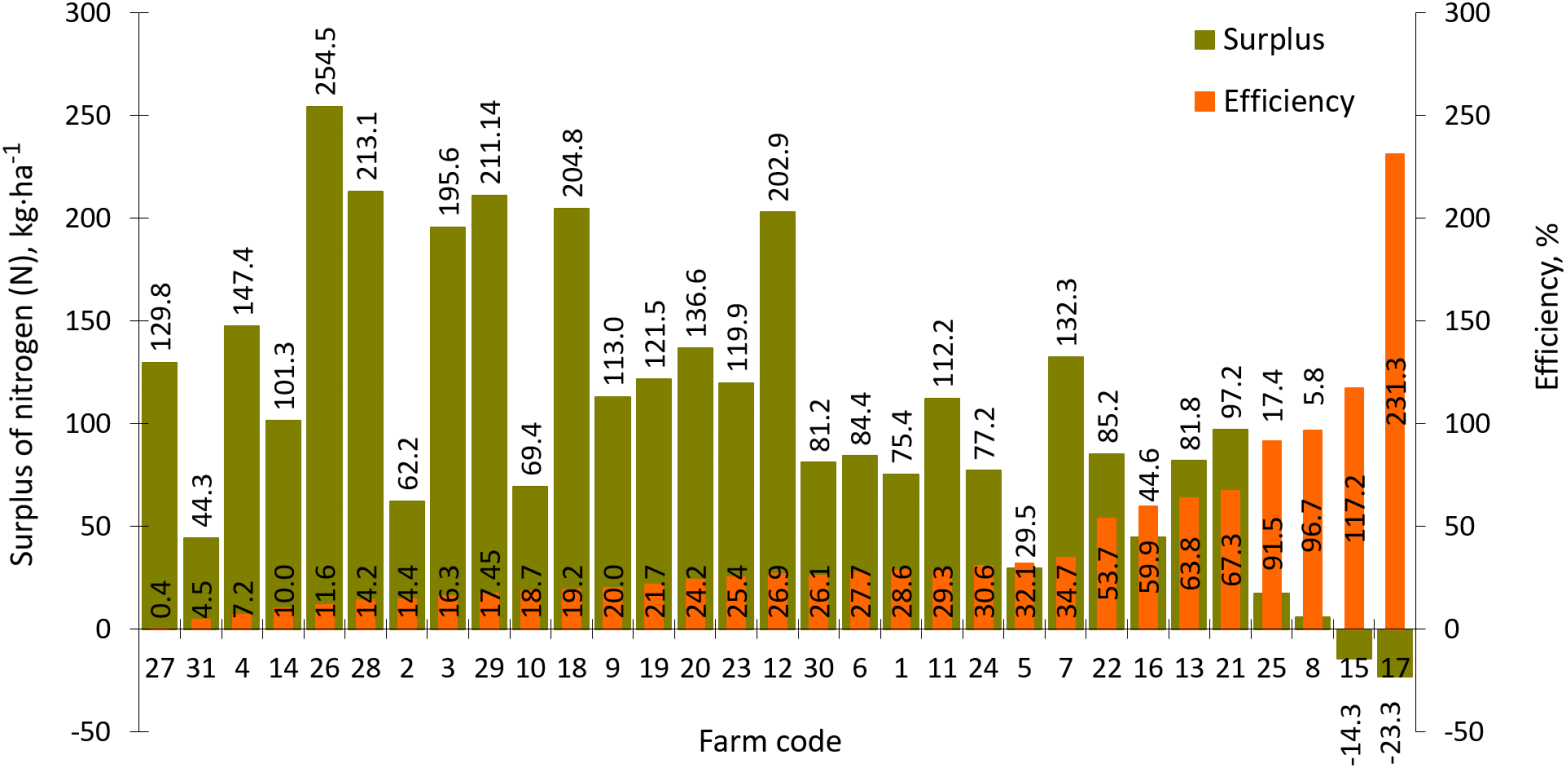
Surplus and efficiency of nitrogen (N) use in farms participating in the WaterPUCK project.

Nitrogen balances on the analyzed farms ranged from −23.3 to 254.5 kg N·ha^−1^ AL while N use efficiency ranged from 0.40% to 231.3% (Fig. 10). The lowest efficiency, 0.4%, was observed in the horse-breeding farm (Code 27) while the highest level, 231.3%, was recorded in the sole plant production farm (Code 17). The average nitrogen surplus in all 31 farms was 120.6 kg N·ha^−1^ AL while efficiency of this component use was 31.8%.

**Figure 11:**
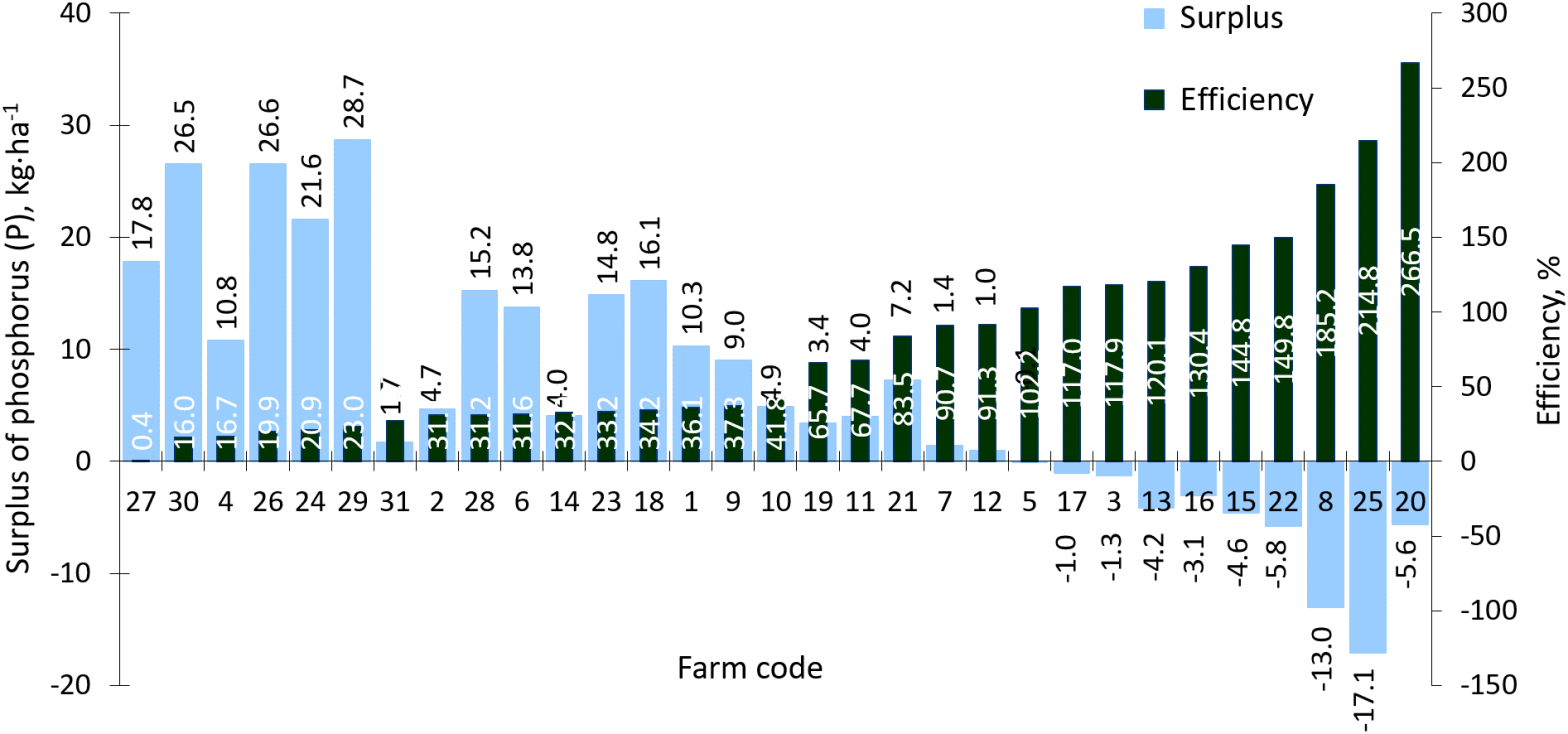
Surplus and efficiency of phosphorus (P) use in farms participating in the WaterPUCK project.

In the case of phosphorus, the average P surplus/deficit value for all farms was 5.0 kg P·ha^−1^ AL (Fig. 11) with a farm range of −17.11 to 28.7 kg P·ha^−1^ AL (Fig. 11). The average P use efficiency was 66.2% while on farms ranged from 0.4% to 266.5%.

**Figure 12:**
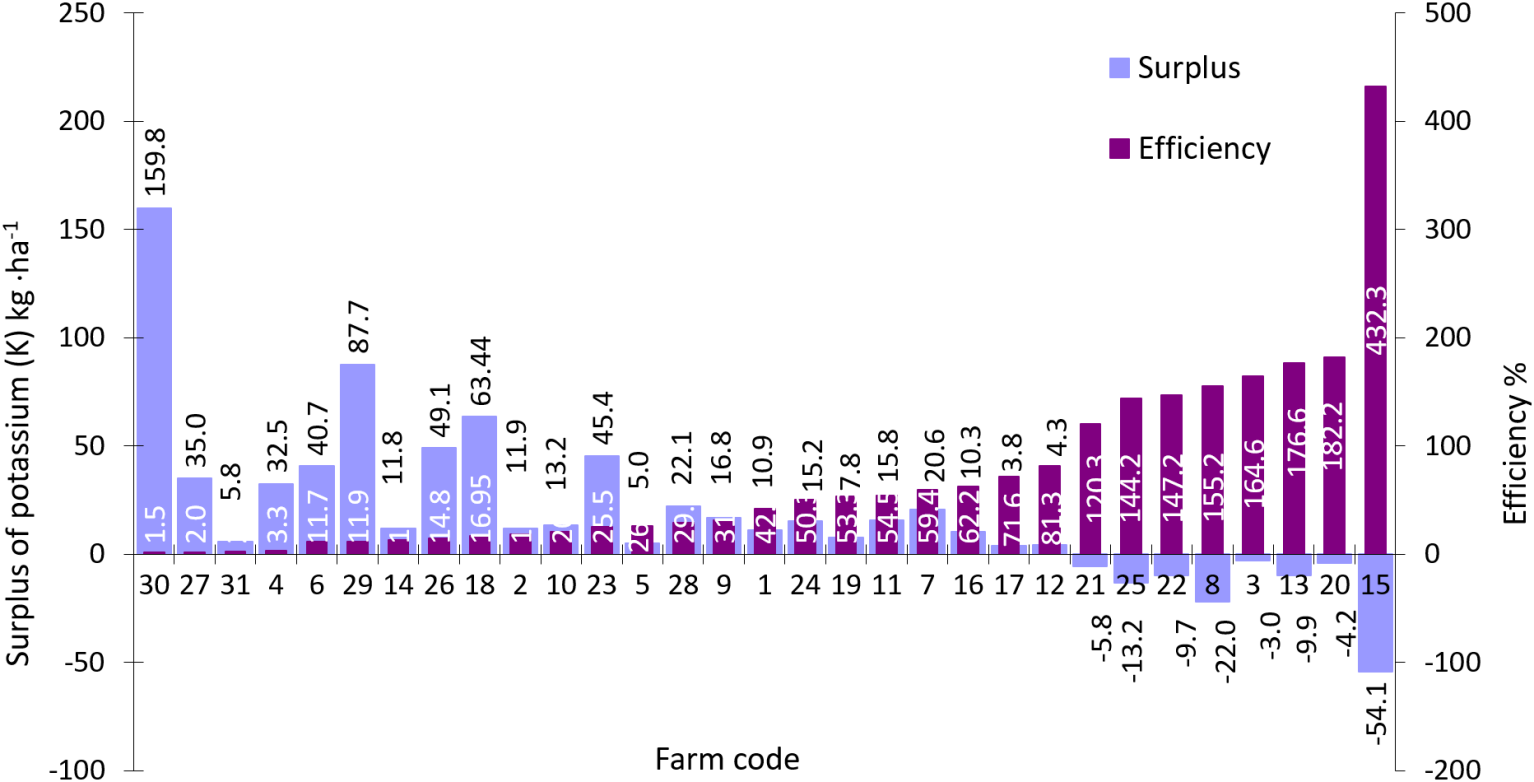
Surplus and efficiency of phosphorus (K) use in farms participating in the WaterPUCK project.

Potassium balances and use efficiency on study farms ranged from −54.1 to 159.8 kg K·ha^−1^ AL and from 1.5% to 432.3%, respectively (Fig. 12). The average K surplus value was 10.8 kg K·ha^−1^ AL while average K use efficiency was 62.2%.

With regard to all agricultural holdings, in general structure of N inputs the largest amounts came from mineral fertilizers (65%) and purchased concentrated fodder (17.7%). The next order was as follows: legumes (6.3%), atmospheric precipitation (5.1%), soil microorganisms (4.2%) and others (0.6%). In structure of N outputs, the largest amount was nitrogen sold in plant products (62.3%) while the remaining N part (37.7%) was sold in animal products.

In P balance, the order of the largest proportions of P input was mineral fertilizers (63%), purchased concentrated fodder (32.7%), atmospheric precipitation (2.5%), others (1.6%), while P was output in sold plant (57.4%) and animal products (32.7%).

As with N and P, in K balance the order of inputs was mineral fertilizers (79.4%), purchased concentrated fodder (10.6%), atmospheric precipitation (9.1%) and others (0.9%). In structure of K outputs sold, plant products (77.4%) predominated over animal products (22.6%).

## Discussion

Impact of agricultural farms on the environment of the Puck Commune caused by dispersion of fertilizer components, was determined by a set of natural and anthropogenic factors conditioning the activities of these farms. Undoubtedly, the most important factors were those that concerned the use of mineral fertilizers. Nitrogen fertilizers consumption in the tested farms was higher than average usage across Poland and in the Pomeranian Voivodeship, compared to the lesser consumption of potassium fertilizers (Table 3). Phosphorus fertilizers consumption was higher than in the Pomeranian Voivodeship, but lower compared to the entire country. Most of the farms of the Puck Commune used N fertilizers in doses of 50–100 (35.5%, *n* = 11) and 100–150 kg N·ha^−1^ AL (the same) while P fertilizers in doses of 10–15 kg P·ha^−1^AL (32.3%, *n* = 10) and 5–10 kg P·ha^−1^ AL (25.8%, *n* = 8). In case of K fertilizers, the largest two groups of farms (35.5%, *n* = 11) used them in doses between 0–20 and 20–40 kg K·ha^–1^ AL. N:P_2_O_5_:K_2_O ratio in average fertilizer dose for all farms was 1.0:0.19:0.24 (what means that for every one kg of N only 0.19 kg of P_2_O_5_ and 0.24 kg of K_2_O were applied). These proportions may raise some doubts in the light of the general recommendations of crop fertilization. According to them, 1.00:0.50:0.98 proportions are recommended for fertilization that is sustainable for field crops in Polish soil conditions and 1.00:0.46:0.68 for permanent grassland (Kucharska, Staśkiewicz & Wronka, 1996). It should be also emphasized that in conditions of wrong N:P:K ratios in fertilizers usage there may occur some disturbances in process of N acquirement by plants and increased losses of this component, causing environmental hazards.

**Table 3:** Consumption of mineral fertilizers (calculated on the pure ingredient) per one ha of agricultural land in the marketing year of 2016/2017.

Area	Mineral fertilizers consumption. kg·ha^−1^ AL			
	Total (NPK)	Nitrogen (N)	Phosphorus (P)	Potassium (K)
Poland*	121.6	79.4	10.3	31.9
Pomeranian Voivodship*	121.1	82.8	8.8	29.5
Farms surveyed—average	147.1	114.9	9.3	22.9

**Note:**

* Central Statistical Office of Poland (CSO) (2018).

Considering the environmental aspects of fertilizer usage, it can be concluded that the majority of farms in the Puck Commune used the correct approach in mineral fertilizers management (e.g., dividing doses, not using fertilizers in high-risk conditions, observing rules for fertilizer use on slopes, no fertilizers in proximity to surface water, keeping agro-technical practices records). However, in most of farms there were natural conditions that could create increased fertilizers losses during their application, especially in which arable lands were located in steep-slope areas (more than 60% of all farms). On such plots, surface runoff could be formed, delivering nutrients from land to watercourses and water reservoirs. Therefore, this could lead to their eutrophication (Andraski & Bundy, 2003; Miller et al., 2011). Therefore, the higher fertilizers doses were used, the greater could be the loss of nutrients by surface runoff (Thayer, 2011; Smith, Jackson & Pepper, 2001).

NP and K in mineral fertilizers constituted the largest shares in total components input brought onto the analyzed farms from outside (on average, 65.0%, 63.0% and 79.4%, respectively). Moreover, the relationship between N, P and K content in mineral fertilizers and their surplus generated by farms has a strong positive correlation (Table 4–6).The average N and K surplus had also a statistically significant positive impact on purchased concentrated fodder while in case of average P surplus this relationship did not occur. These two sources frequently determine the N surplus size estimated by the “At the farm gate” method (Pietrzak, 2009; Kupiec, 2011).

**Table 4:** The relationship between the surplus of N and selected factors.

	Surplus N (kg·ha^−1^)	Efficiency (%)	Nitrogen in mineral fertilizers (kg·ha^−1^)	Nitrogen in feeds (kg·ha^−1^)	N share in the sold animal production (%)	N share in the sold plant production (%)
Surplus N (kg·ha^−1^)	1.00					
Efficiency (%)	−0.58	1.00				
Nitrogen in mineral fertilizers (kg·ha^−1^)	0.57	0.04	1.00			
Nitrogen in feed (kg·ha^−1^)	0.48	−0.18	0.03	1.00		
N share in the sold animal production (%)	0.36	−0.53	−0.20	0.64	1.00	
N share in the sold plant production (%)	−0.36	0.53	0.20	−0.64	−1.00	1.00

**Note:**

Correlation Spearman ranks order, marked (in red) correlations are significant–with *p* < 0.05.

**Table 5:** The relationship between the P surplus and selected factors.

	Surplus P (kg·ha^−1^)	Efficiency (%)	Phosphorus in mineral fertilizers (kg·ha^−1^)	Phosphorus in feeds (kg·ha^−1^)	P share in the sold animal production (%)	P share in the sold plant production (%)
Surplus P (kg·ha^−1^)	1.00					
Efficiency (%)	−0.91	1.00				
Phosphorus in mineral fertilizers (kg·ha^−1^)	0.57	−0.43	1.00			
Phosphorus in feed (kg·ha^−1^)	0.33	−0.10	−0.04	1.00		
P share in the sold animal production (%)	0.44	−0.44	−0.12	0.51	1.00	
P share in the sold plant production (%)	−0.44	0.44	0.12	−0.51	−1.00	1.00

**Note:**

Correlation of the Spearman ranks order, marked (in red) correlations are significant–with *p* < 0.05.

**Table 6:** The relationship between the K surplus and selected factors.

	Surplus K, kg·ha^−1^	K efficiency (%)	K in mineral fertilizers (kg·ha^−1^)	K in feeds (kg·ha^−1^)	K in sold animal products (kg·ha^−1^)	K in sold plant products (kg·ha^−1^)
Surplus K (kg·ha^−1^)	1.00					
K efficiency (%)	−0.81	1.00				
K in mineral fertilizers (kg·ha^−1^)	0.65	−0.41	1.00			
K in feed (kg·ha^−1^)	0.36	−0.19	0.01	1.00		
K in sold animal products (kg·ha^−1^)	0.26	−0.06	0.02	0.52	1.00	
K in sold plant products (kg·ha^−1^)	−0.52	0.62	0.14	−0.40	−0.48	1.00

**Note:**

Correlation of the Spearman ranks order, marked (in red) correlations are significant–with *p* < 0.05.

In addition to purchased fertilizers and concentrated fodder, the factors that had a significant impact on the results of N, P and K balances were sold plant products as well as sold animal products—it was an inverse relationship. In the N and P cases, there were also significant positive correlations between surpluses of these nutrients and their outputs in sold animal products. With regard to K balances, no such relationships were found.

The average N surplus in farms of the Puck Commune was 120.6 kg N·ha^−1^ AL while the average P surplus was at a level of −5.0 kg P·ha^−1^ AL (values of these indicators were characterized by a considerable variety among the surveyed farms). According to various authors works (Godinot et al., 2015; Olofsson, 2015), the levels of N and P surplus determined using farm scale nutrient balance are closely related to their business profile—the largest NP surplus are generated on farms focused on animal production.

The broad majority of farms in the Puck Commune (80.6%, *n* = 25) was focused on livestock production, in particular, milk and beef (48%, *n* = 12), only pork (24%, *n* = 6), only beef (8%, *n* = 2), beef and pork production (24%, *n* = 6) and horse breeding (4%, *n* = 1).Comparing study farms average N surplus, it can be concluded that its value was smaller in relation to a similar category of French farms (Table 7), while compared to Swedish farms, it was at comparable level (Table 8). Comparable in level to farms in Sweden was also an average P surplus calculated for all surveyed farms in the Puck Commune. In view of the fact that in Sweden huge attention to reducing the losses of nutrients from agriculture is paid, especially due to need of counteracting Baltic Sea eutrophication, it seems that N and P surplus generated by farms of the Puck Commune can be considered acceptable in the context of their impact on the environment.

**Table 7:** Mean surplus N and N-efficiency in nine farming system categories in France (based on: Godinot et al., 2015).

	Farming system categories								
	Beef cattle	Beef cattle and crops	Beef cattle and pigs	Crops	Crops and milk	Milk	Milk and pigs	Pigs	Poultry
Number of farms	47	35	13	24	53	299	36	30	20
Surplus N (kg N·ha^−1^ AL)	228	128	448	141	124	245	420	852	377
N-efficiency (%)	11.6	30.4	17.5	41.7	27.9	16.9	21.9	23.5	26.8

**Table 8:** Farm gate balances of conventional farms in southern Sweden (based on: Olofsson, 2015).

	Type of farms		
	Crop	Dairy	Pig
Number of farms	965	976	204
Surplus N (kg N·ha^−1^ AL)	45	143	104
Surplus P (kg P·ha^−1^ AL)	−1.4	4.7	7.6

The average surplus of K—a component regarded as neutral for the environment—in study farms was 10.8 kg K·ha^−1^ AL. The level of this surplus was 28% lower than in K balance found in other researches undertaken in Poland on a comparable group of farms (in terms of number of farms and their specialization of production), but located in a region with more intensive agriculture (Kupiec, 2015).

With regard to the presented results of nitrogen, phosphorus and potassium balance, it should be noted that they might be affected by some uncertainty associated to method of obtaining results for their preparation, based on interviews with farmers. Therefore, it is right to postulate that keeping records on agro-technical practices or nutrient booking containing necessary information for balance sheets preparation should be implemented (in particular records on purchased fertilizers and concentrated fodder as well as sold agricultural products) (Kupiec & Zbierska, 2008). Apart from purely cognitive values of nutrient balance results, they have an educational significance in shaping farmers’ awareness. This meaning is widely articulated in many sources and can be expressed in the form of the following opinions and statements:

- The “At the farm gate” nutrient balance method is a basic and simple way to increase knowledge and farmers’ awareness about nitrogen, phosphorus and potassium flow–to and from a farm,–creating a starting point for discussion on how to use these components efficiently on farm scale and on impact of NPK and their incomplete use on farm economics as well as the environment (Nilsson, 2013);
- Nutrient balance enables farmers to easily review NPK flow at farm gate level by calculating the amount of nutrient imported and exported to the farm. Thanks to that, a well-prepared nutrient balance can help the farmer to evaluate and improve their nutrient management which can contribute to lower operating costs of the farm by showing the actual amount of nutrients needed for production (Nutrient balance; Farmgate Nutrient Balance Help file PLANET version 3.0, 2010);
- Farm gate nutrient balances are a useful tool to compare farms and farm systems as well as to identify high-risk areas where a lot of nutrients is gathered and hotspots for nutrient emissions (Ramnerö, 2015).

By calculating the nutrient balances at farm gate level, based on the principles of farmers’ voluntary participation and through their dialogue with the advisory institutions, an agreement may be achieved—in order to reduce NPK surpluses and to increase farm profit (Olofsson, 2014).

In the light of the above, preparation of tool called integrated agriculture calculator—CalcGosPuck within the WaterPUCK project is well grounded and fully justified. Its dissemination may contribute to broadening farmers’ knowledge on correct nutrient management and fertilizer on farm scale and thus reduce environmental pressure exerted by agricultural activities.

## Conclusion

The environmental impact of study agricultural holdings in the Puck Commune (which can be taken as *representatives* of the *entire collectivity* in this commune) was mainly related to the amount of mineral nitrogen and phosphorus fertilizers consumption in these farms as well as practices and conditions of their use. The mean N fertilizers consumption per one ha of agriculture land in the study area was significantly higher in comparison to their average unit usage in Poland, while the mean consumption of P fertilizers was slightly lower than the national average. At the time of application these fertilizers, the recommendations for reducing their environmental impact were considered. The amount of purchased N, P and K fertilizers had a significant impact on the results of nutrient balances estimated by the “At the farm gate” method. The results of nutrient balances showed, in particular, that average N, P and K surplus generated by the analyzed farms ranged within the respective levels of 120.6 kg N, 5.0 kg P and 10.8 kg K·ha^−1^ AL. Comparing nutrient surplus amount in agricultural holdings of the Puck Commune to similar farms and farm systems, for an example, in countries with well-developed agriculture, such as France and Sweden, average N and P surplus in study area can be assessed as moderate while average K surplus as being in the range of its average values typical for farms in Poland.

Notwithstanding the above, the results of estimated NPK balance well showed their practical dimension. In this regard, it should be indicated that estimating N, P and K values in a nutrient balance can lead to many practical conclusions helping to reduce the impact of agricultural production on the environment and to improve the farming economy. An example of the latter would be the results of more effective use of nutrients on a farm and lower expenditures on fertilizers and feeds. Therefore, knowledge on how to estimate nutrient balances should be more widely disseminated, especially among farmers and agricultural advisors. Helpful role in this area can play program developed within the WaterPUCK project called “Integrated agriculture calculator–CalcGosPuck.” CalcGosPuck works as an independent application to calculate the pollution emission from agricultural holdings to the environment, including surface and groundwater, but it also can serve to calculate the nutrients’ distribution over agricultural areas.

## Supplemental Information

10.7717/peerj.6478/supp-1 Supplemental Information 1 Data for basic information on farms participating in the WaterPUCK project.

10.7717/peerj.6478/supp-2 Supplemental Information 2 Data for type and area of arable land or grassland in farms participating in the WaterPUCK project.

10.7717/peerj.6478/supp-3 Supplemental Information 3 Data for dates due of liquid natural fertilizers use (manure, slurry) on permanent meadows and dates due of solid manure use on arable lands in farms participating in the WaterPUCK project.

10.7717/peerj.6478/supp-4 Supplemental Information 4 Data for consumption of nitrogen (N), phosphorus (P) and potassium (K) mineral fertilizers in individual farms participating in the WaterPUCK project.

10.7717/peerj.6478/supp-5 Supplemental Information 5 Data for surplus and efficiency of nitrogen (N) and phosphorus (P) use in farms participating in the WaterPUCK project.

10.7717/peerj.6478/supp-6 Supplemental Information 6 Data for surplus and efficiency of potassium (K) use in farms participating in the WaterPUCK project.

10.7717/peerj.6478/supp-7 Supplemental Information 7 The structure of the surveyed households by size based on the size scale in Polish FADN.

10.7717/peerj.6478/supp-8 Supplemental Information 8 Share of the land with the individual categories–in the area covered by the study in farms participating in the WaterPUCK project.

10.7717/peerj.6478/supp-9 Supplemental Information 9 Method of silage storing in farms participating in the WaterPUCK project.

10.7717/peerj.6478/supp-10 Supplemental Information 10 Dates due of solid manure use on arable lands in farms participating in the WaterPUCK project.

10.7717/peerj.6478/supp-11 Supplemental Information 11 Dates due of liquid natural fertilizers use (manure, slurry) on permanent meadows in farms participating in the WaterPUCK project.

## Additional Information and Declarations

### Data Availability

The following information was supplied regarding data availability: The raw data are available in the Supplemental Files.
