# Supplementary figures and images for: Impact of agricultural farms on the environment of the Puck Commune: Integrated agriculture calculator—CalcGosPuck
Source: PeerJ. 2019 Feb 19;7:e6478. doi: 10.7717/peerj.6478 (PMC6385684; doi:10.7717/peerj.6478)

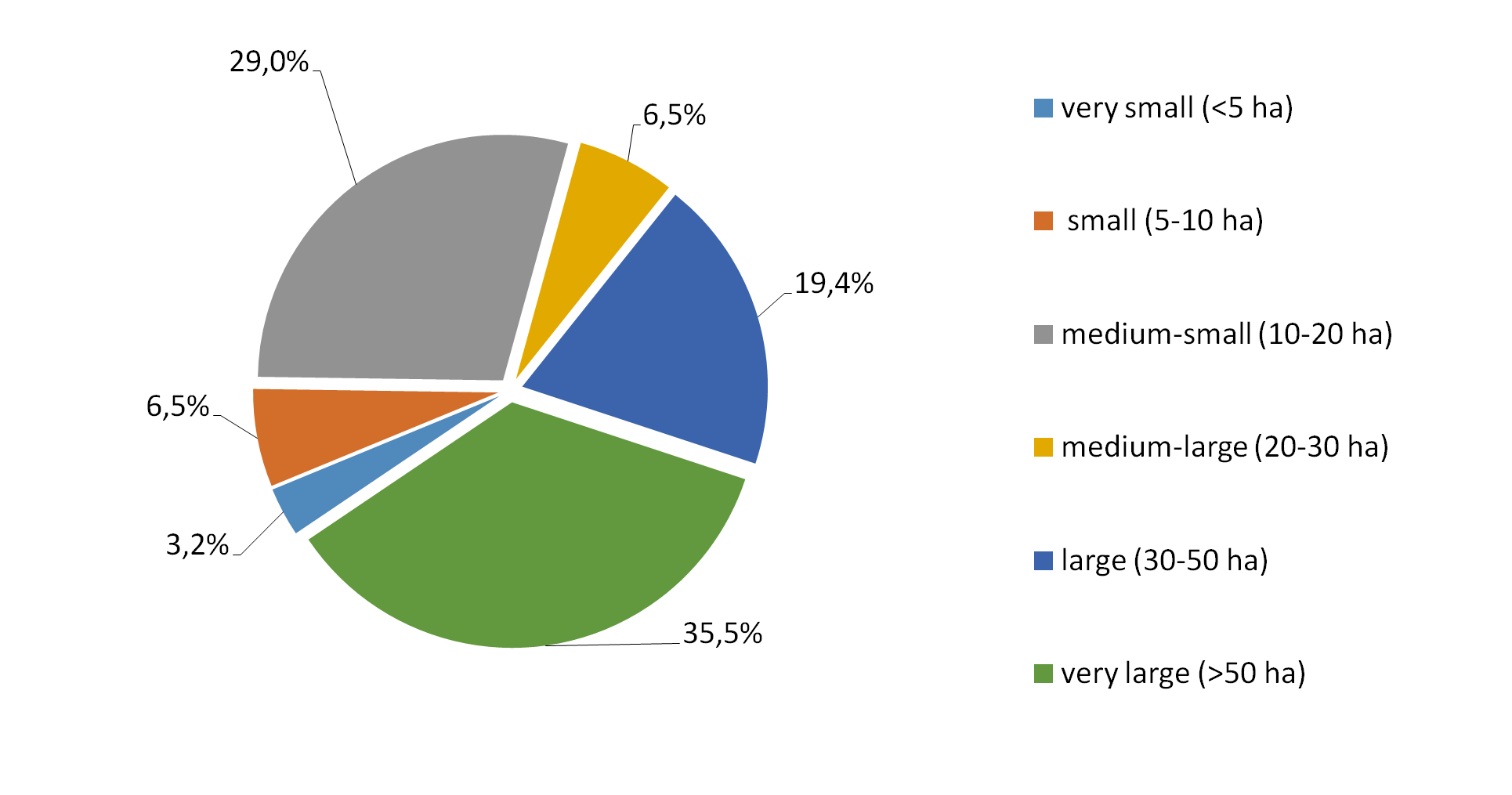

Supplement: Supplemental Information 7 [file peerj-07-6478-s007.png]

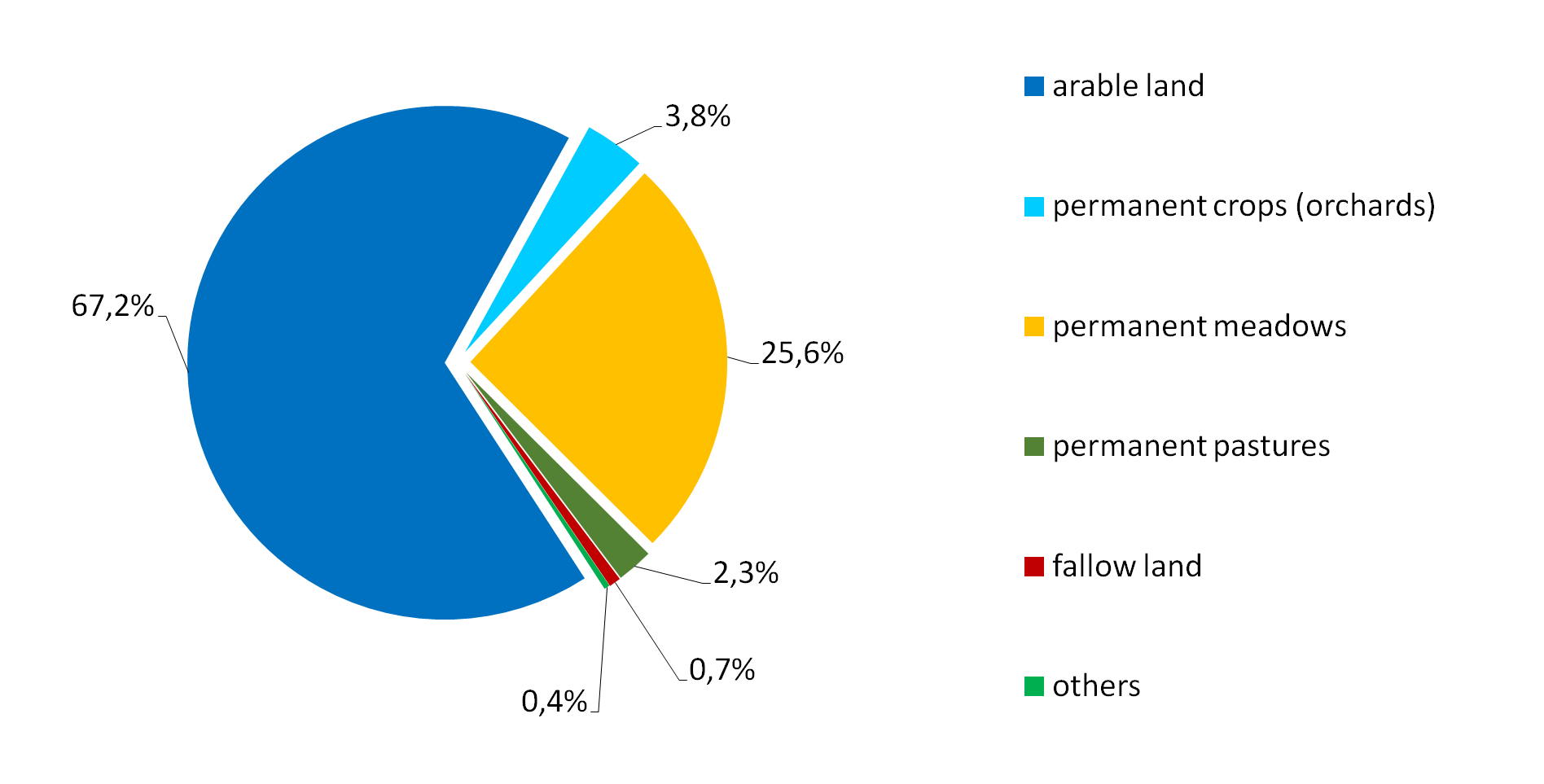

Supplement: Supplemental Information 8 [file peerj-07-6478-s008.png]

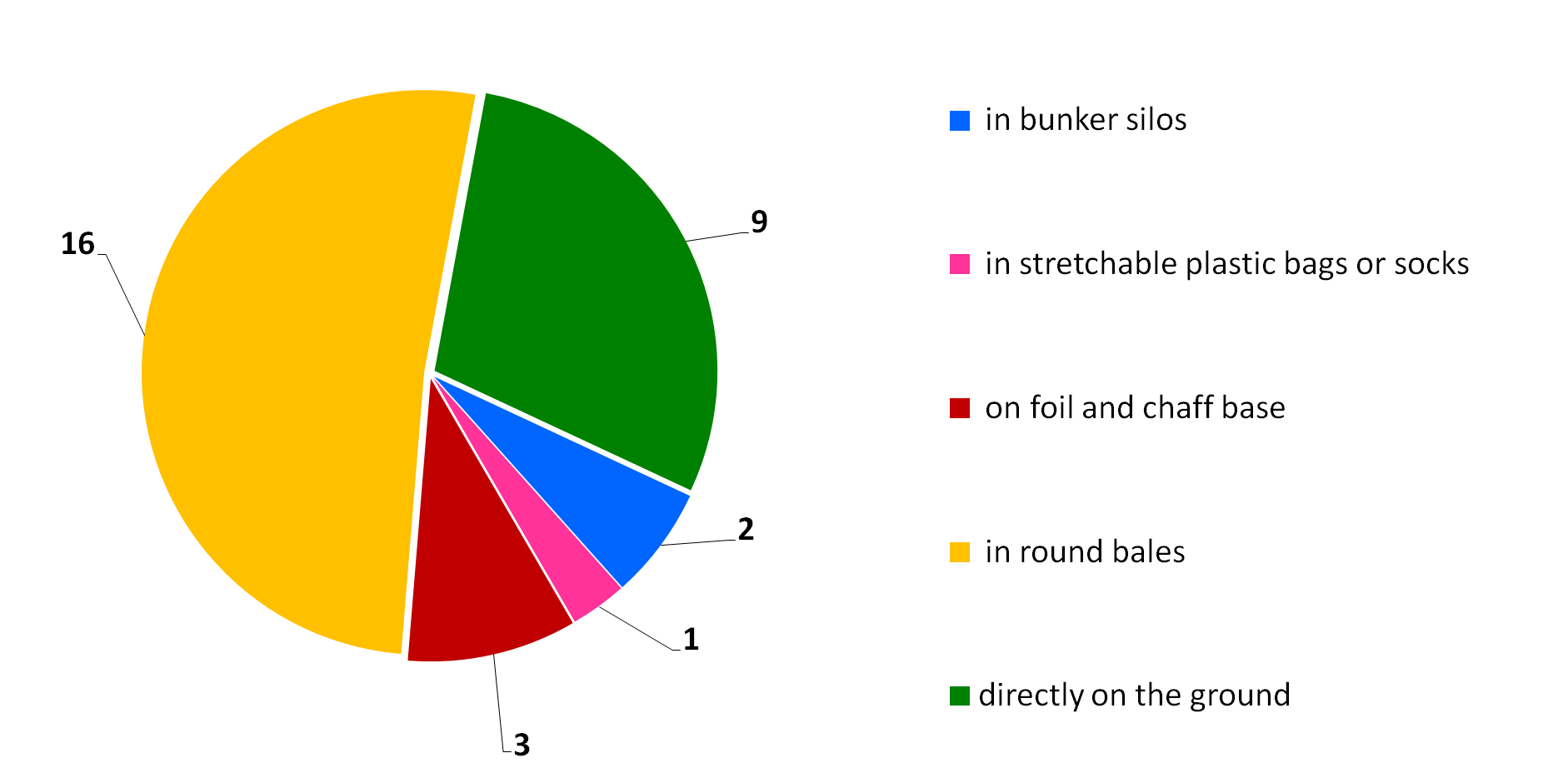

Supplement: Supplemental Information 9 [file peerj-07-6478-s009.png]

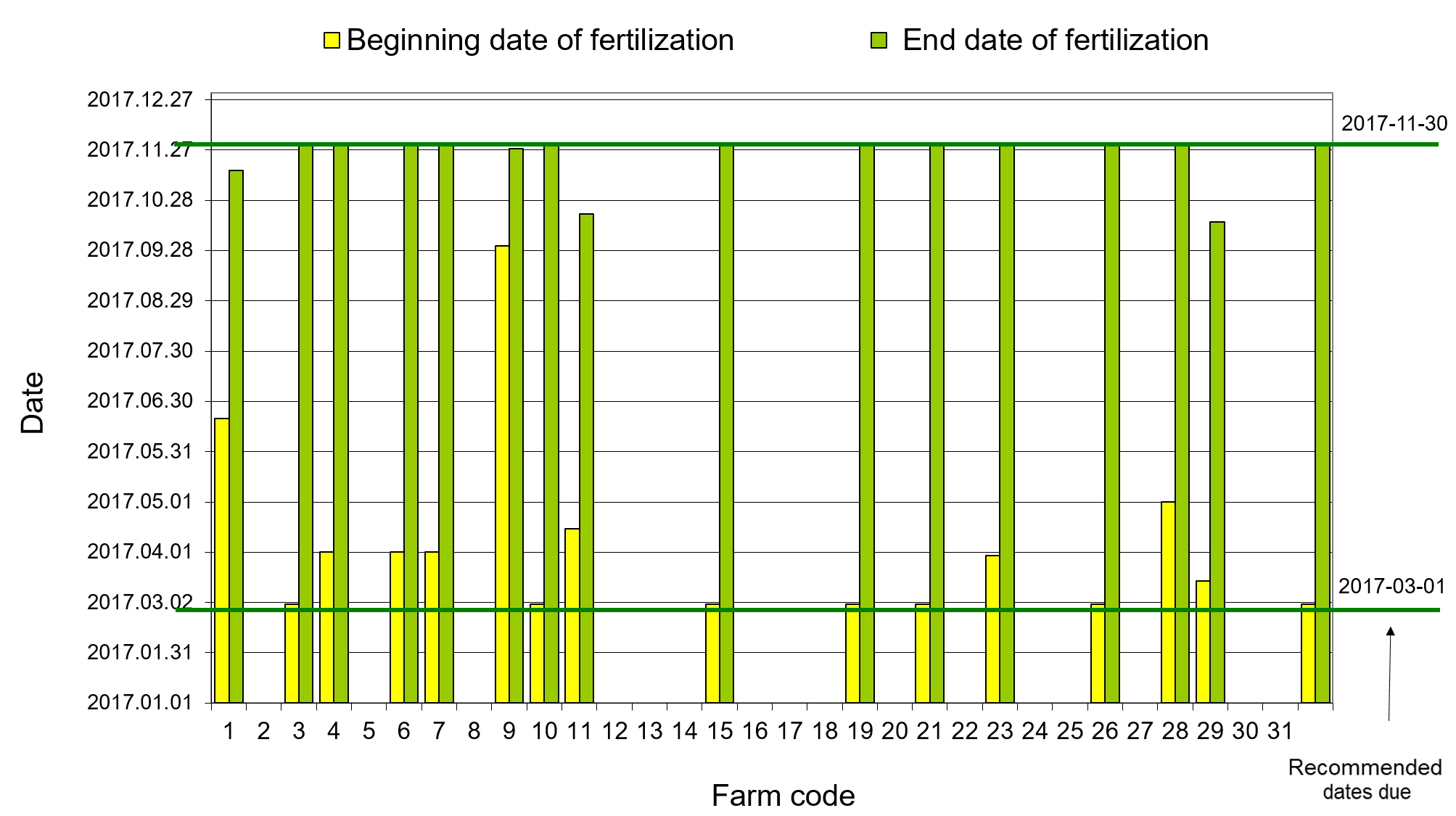

Supplement: Supplemental Information 10 [file peerj-07-6478-s010.png]

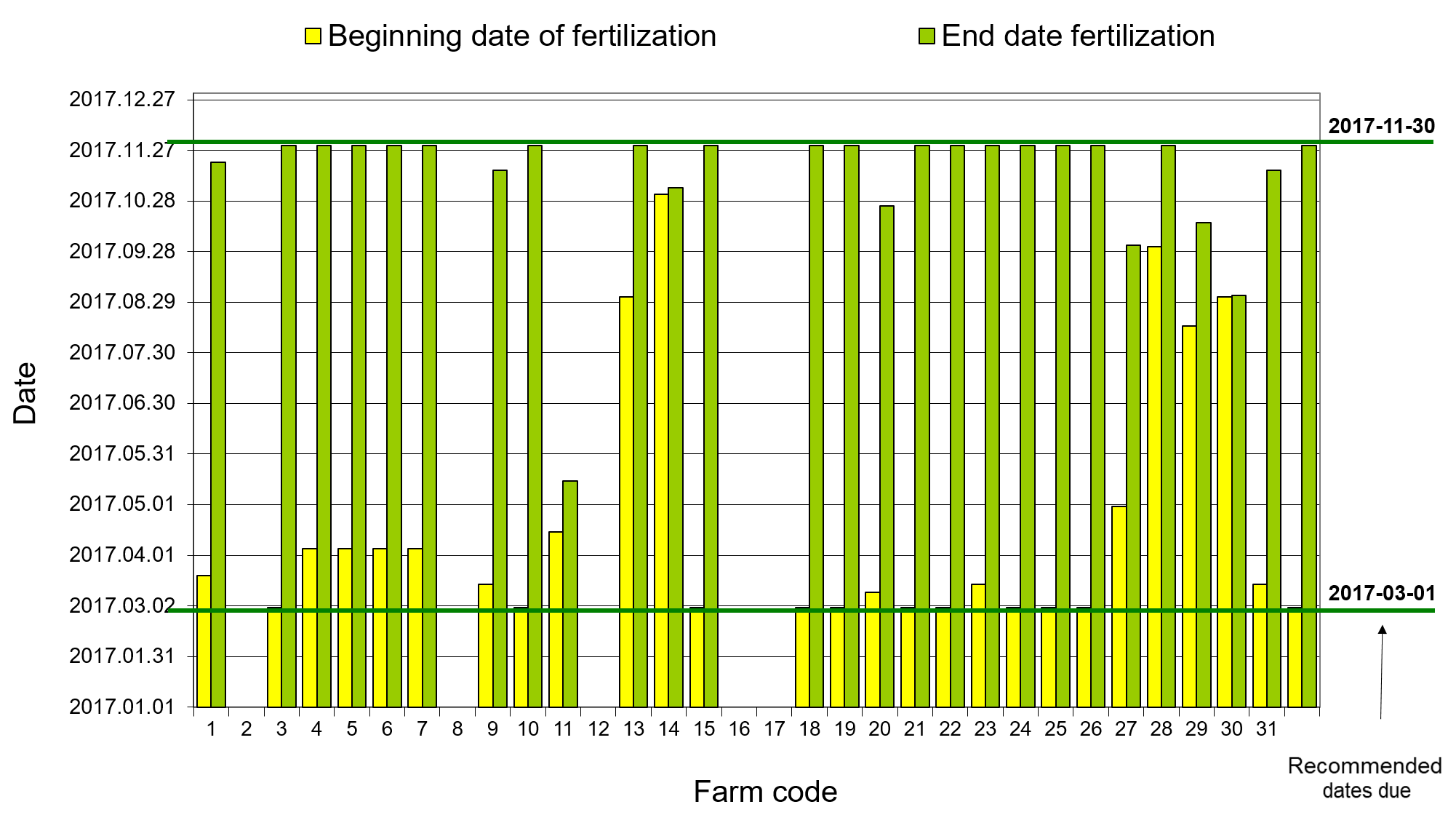

Supplement: Supplemental Information 11 [file peerj-07-6478-s011.png]
